# CD44/ERM/F‐actin complex mediates targeted nuclear degranulation and excessive neutrophil extracellular trap formation during sepsis

**DOI:** 10.1111/jcmm.17231

**Published:** 2022-02-11

**Authors:** Yiming Shao, Linbin Li, Lu Liu, Yunxi Yang, Jiamin Huang, Dongdong Ji, Yuying Zhou, Yi Chen, Zhechen Zhu, Bingwei Sun

**Affiliations:** ^1^ Department of Burns and Plastic Surgery Affiliated Suzhou Hospital of Nanjing Medical University Suzhou Jiangsu Province China; ^2^ School of Medicine Jiangsu University Zhenjiang Jiangsu Province China

**Keywords:** CD44, degranulation, myeloperoxidase, neutrophils extracellular trap

## Abstract

Neutrophils release neutrophil extracellular traps (NETs) to capture and kill pathogens, but excessive NET release can damage the surrounding tissues. Myeloperoxidase (MPO) and neutrophil elastase (NE) are thought to be important in promoting histone depolymerization and DNA breakage in the nucleus. However, the detailed path by which MPO and NE enter the nucleus is unknown. In the present study, we observed that delayed fusion of azurophilic granules with the nuclear membrane 15–20 min after extracellular degranulation in activated neutrophils. In a subsequent experiment, we further demonstrated that this fusion leads to MPO entry into the nucleus and promotes nuclear histone depolymerization and DNA breakage, a process called ‘targeted nuclear degranulation’. This process can be effectively inhibited by dexamethasone and accompanied by the continuous low levels of MPO in the nucleus after PMA stimulation. Meanwhile, we found that ‘targeted nuclear degranulation’ is dependent on the CD44 translocation and subsequent redistribution of CD44 / ERM (Ezrin/Radixin/Moesin) / F‐actin complexes, which guides the movement of azurophilic granules towards the nucleus. Application of ERM phosphorylation inhibitors and importin activity inhibitors significantly reduced the complexes formation and redistribution. Taken together, these findings indicate for the first time that delayed ‘targeted nuclear degranulation’ after neutrophil activation is a key mechanism of NET formation. CD44/ERM/F‐actin complex mediates this process, which providing targets with promising prospects for the precise regulation of NET formation.

## 1 INTRODUCTION

Neutrophils play an important role in the process of infection.^1^ After these cells first reach the site of infection, neutrophils eliminate pathogens through phagocytosis, degranulation and reactive oxygen species (ROS) formation.^2^, ^3^ However, proteolytic enzymes released by activated neutrophils can destroy surrounding tissue. Sepsis is a systemic inflammatory response that leads to the extensive activation of neutrophils, which release a large number of active cytokines and exacerbate organ dysfunction.^4^ Therefore, inhibiting excessive neutrophil activation in sepsis can improve the condition of septic patients.

Neutrophil extracellular traps (NETs) released from activated neutrophils are composed of extracellular web‐like DNA and antimicrobial proteins, including myeloperoxidase (MPO).^5^ We found significantly higher levels of histones in the plasma of patients with sepsis than in controls. A variety of enzymes, including MPO and neutrophil elastase (NE), adhere to NETs and continue to exert antimicrobial effects.^5^, ^6^ One of our studies also showed that the release of enzymes such as MPO and heparin‐binding protein (HBP) is an important cause of vascular endothelial damage. A large number of neutrophils release nets during sepsis. The enzymes carried by these NETs cause persistent local tissue damage and may be responsible for multiple organ dysfunction.

The mechanism of NET formation remains unclear. It is believed that nuclear histone depolymerization is necessary for DNA release.^7^ Several enzymes, including NE, MPO and peptidylarginine deiminase 4 (PAD4), are involved in the depolymerization of histones in the nucleus.^6^, ^8^, ^9^ However, the process by which these enzymes are transferred from the cytoplasm or even within the granule to the nucleus remains unclear. Metzler, K.D. et al. showed that ROS trigger NE translocation from granules into the cytoplasm, activating the proteolytic activity of NE in an MPO‐dependent manner, and eventually, NE is transferred into the nucleus.^6^ Gasdermin D (GSDMD) pores are localized on neutrophil azurophilic granules and the nucleus and may act as a pathway for NE translocation into the nucleus.^10^ However, how MPO (a large‐molecular‐weight protein) is transferred to the nucleus has not been reported. The degranulation of neutrophils consists of the translocation of granules to the plasma membrane and their subsequent fusion with the plasma membrane. The binding of vesicle‐associated membrane proteins (VAMPs) to syntaxin 4 (sy4) initiates fusion of the cellular and granule membranes.^11^, ^12^ After the granules fuse with the plasma membrane, granule proteins are released via exocytosis and participate in innate immune responses. Several factors influence neutrophil degranulation, including the expression of proteins that mediate membrane fusion, the polymerization/depolymerization of actin and cellular Ga^2+^ levels.^13^ Surprisingly, we found that azurophil granules could fuse with the nuclear membrane and release MPO into the nucleus through degranulation to the nucleus, which in turn promotes NET formation. We named this process targeted nuclear degranulation. We also found that changes in actin appear to have a large effect on targeted nuclear degranulation. Interestingly, we found that the initiation of targeted nuclear degranulation occurred significantly later than degranulation to the extracellular space and that this effect was associated with CD44 translocation to the nucleus.

CD44 is a widely expressed type I transmembrane protein that is the major receptor for hyaluronic acid (HA). CD44 plays an important role in neutrophil adhesion and migration.^14^ Its binding to ERM anchors the cytoskeleton to the cell membrane, which also plays an important role in maintaining cell morphology and motility.^15^, ^16^ CD44 is cleaved and shed into the cytosol, and its intracellular domain (ICD) can be transferred to the nucleus as a signal to participate in the regulation of gene transcription.^17^ It has also been reported that full‐length CD44 can be transferred to the nucleus,^18^ which also provides a theoretical basis for our study.

The present findings reveal a novel mechanism of NET formation. Several of the important targets identified are expected to serve as therapeutic targets to reduce NET formation and mitigate organ damage under septic conditions.

## 2 MATERIALS AND METHODS

### 2.1 Ethical statement

This study was approved by The Medical Ethical Committee of Nanjing Medical University. For experiments involving human blood samples, signed informed consent was obtained from all patients and healthy volunteers. Blood samples were taken from the cubital veins of patients and healthy donors. All the experimental methods were carried out in accordance with the approved guidelines. All experimental procedures involving mice were carried out in strict accordance with the recommendations in the Guide for the Care and Use of Laboratory Animals of the National Institutes of Health and State Key Laboratory of Pathogens and Biosecurity of the Institute of Microbiology and Epidemiology.

### 2.2 Neutrophil extraction

Blood samples from patients with sepsis were obtained from the ICU of Suzhou Municipal Hospital, and blood samples from healthy individuals were obtained from the medical examination centre. The aforementioned blood donors signed an informed consent form. The diagnosis of sepsis in the patients was confirmed according to Sepsis 3.0.

Neutrophils were isolated and purified using magnetic beads (STEMCELL Technologies, #18103, #19666). Briefly, 50 µl of liquid A and 50 µl of liquid B (magnetic beads) were added to each 1‐ml blood sample, mixed and allowed to stand for 5 min. Stem buffer diluted 1:1 was added to the blood sample, which was then placed in a magnetic rack and allowed to stand for 10 min. The supernatant was aspirated, 50 µl of solution B was added to each 1 ml of supernatant, and the mixture was again placed in the magnetic rack for 5 min. The supernatant was aspirated and again placed in the magnetic rack for 5 min. The supernatant was aspirated and centrifuged at 400 × *g* for 5 min to obtain neutrophils. Neutrophils were extracted and stored in RPMI‐1640 medium (Gibco, Canada) containing 10% FBS.

### 2.3 CLP model

Male C57BL/6 mice (8 weeks old, Suzhou, China) were maintained at the Animal Experimental Center of Suzhou Municipal Hospital under a 12‐h light‐night cycle with free access to food and water for at least 1 week before the experiments.

Sepsis was induced by caecal ligation and puncture (CLP) in the C57BL/6 mice. The mice were anaesthetized with 10% chloral hydrate by intraperitoneal injection (300 mg/kg body weight), the cecum was exposed by a middle abdominal incision, and the end of the cecum was punctured with a 22‐gauge needle after the cecum had been ligated. After the abdomen was closed, normal saline was injected intraperitoneally. Sham‐operated mice and CLP mice were sacrificed 24 h after surgery. The lungs, liver, spleen and pancreas were removed and fixed in 4% paraformaldehyde.

### 2.4 Neutrophil chemotaxis model

Neutrophil migration was assayed using an agarose neutrophil chemotaxis model. To prepare agarose gels, an agarose solution was mixed with medium consisting of 50% HBSS (containing Ca^2+^ and Mg^2+^) and 50% RPMI‐1640 medium (containing 20% heat‐inactive FBS). Then, 2.7 ml of the solution was pipetted into a 35‐mm culture dish and cooled at room temperature (RT) until solidification. When the agarose solution had completely solidified, three wells (3 mm in diameter and 2.8 mm apart) were cut in the gel. The middle well was filled with 10 µl of the chemoattractant N‐formyl‐Met‐Leu‐Phe (fMLP, 0.1 µM) (59880‐97‐6; Sigma), and the outer wells were filled with 10 µl of neutrophils (1 × 10^7^/ml). The gels were incubated for 2 h in a 37°C/5% CO_2_ incubator. The chemotaxis distance was observed using an Olympus IX71 microscope under a fourfold objective.

### 2.5 NET formation

We inoculated 2 × 106 neutrophils per well into a 12‐well plate supplemented with RPMI‐1640 medium containing 10% FCS. The cells were allowed to settle at the bottom of the wells in the 12‐well plates for 30 min before stimulation with 100 nM PMA. SYTOX Green (1:15,000) was added, and NETs were visualized by fluorescence microscopy. Wherever indicated, cells were preincubated with an NE inhibitor (NEi) at 2 μM (MCE, HY‐17443) or an MPO inhibitor (MPOi) at 1 μM (MCE, HY‐111341) for 30 min before stimulation. Wherever indicated, cells were preincubated with an ERM phosphorylation inhibitor (P‐ERMi) at 10 μM (MCE,HY‐18931A) or an importin‐β inhibitor (Importini) at 30 μM (MCE, HY‐101091) for 30 min before stimulation.

### 2.6 Plasma histone assay

EDTA‐anticoagulated blood was centrifuged at 300 ×g for 10 min at RT, and the top two‐thirds of the plasma was carefully removed and stored at −80°C. Plasma histone levels were determined with a Total Histone H3 Sandwich ELISA Kit (Cell Signaling, #7253) according to the provided protocol. The absorbance at 450 nm was measured within 5 min to determine the histone concentration, and the data were recorded.

### 2.7 Exogenous MPO, NE positioning

Exogenous recombinant MPO (Novoprotein, CS73) and NE (Sigma, E8140) were immunolabelled using anti‐MPO (Abcam, ab208670; 1:100) and anti‐NE (Abcam, ab131260; 1:250) antibodies and then fluorescently labelled with secondary anti‐rabbit fluorescent antibodies. The fluorescently labelled proteins were coincubated with normal neutrophils for 30 min. The neutrophils were then stimulated with PMA for 30 min, and the fluorescence localization of the exogenous proteins was observed under confocal microscopy.

### 2.8 Nucleoprotein and cytoplasmic protein extraction

Nucleoproteins and cytoplasmic proteins were extracted with nuclear and cytoplasmic extraction reagents (Thermo, 78835) according to the supplied protocol. Briefly, the treated cells were washed twice with prechilled PBS and centrifuged at 500 × *g* for 3 min. The cells were resuspended in 200 µl of cytoplasmic extraction reagent I. The suspension was incubated on ice for 10 min, 11 µl of cytoplasmic extraction reagent II was added, incubated on ice for 1 min and then centrifuged at 16,000 ×g for 5 min. The supernatant fraction (cytoplasmic extract) was transferred to a prechilled tube. The insoluble precipitated fraction, containing the crude nuclei, was resuspended in 100 µl of nuclear extraction reagent and incubated on ice for 10 min, followed by centrifugation at 16,000 × *g* for 10 min. The resulting supernatant contained the nucleoproteins. After extraction, the nuclear and cytoplasmic proteins were stored at −80°C until use.

### 2.9 Nuclear membrane protein extraction

Nuclear membrane proteins were extracted with a Nuclear Envelope Protein Extraction Kit (Invent Biotechnologies) according to the provided protocol. Briefly, the collected cells were washed twice with prechilled PBS, resuspended in 0.5 ml of buffer A and centrifuged at 14,000 rpm for 30 s. Then, the supernatant was removed, and the cells were washed once with prechilled PBS. A total of 300 µl of buffer B were used to resuspend the precipitate, which was incubated on ice for 10 min and centrifuged at 8,000 rpm and 4°C for 5 min. After centrifugation at 14,000 rpm and 4°C for 15 min, the supernatant was removed, and the precipitate, which contained the isolated nuclear membrane proteins, was retained. After extraction, the nuclear membrane proteins were stored at −20°C until use.

### 2.10 Western blot analysis

The extracted proteins (nuclear proteins, cytoplasmic proteins and nuclear membrane proteins) were separated by SDS‐PAGE on 10–12% polyacrylamide gels and stained with Coomassie blue. The following primary antibodies were used: anti‐H3 (Abcam, ab1791, 1:1000), anti‐CD63 (Abcam, ab271286, 1:1000), anti‐MPO (Abcam, ab208670, 1:1000), anti‐NE (Abcam, ab131260, 1:1000), anti‐laminB1 (Abcam, ab16048, 1:1000), and anti‐syntaxin4 (Abcam, ab184545, 1:1000), Anti‐CD44 (Abcam, ab189524, 1:1000), anti‐CD44 (Abcam, ab232556, 1:1000), anti‐ERM(Abcam, ab76247, 1:1000) and anti‐pERM(CST, 1:500).

### 2.11 Immunostaining and microscopy

Cells were fixed in 4% paraformaldehyde, permeabilized with 0.1% Triton X‐100, blocked with 5% BSA and stained with the following: anti‐cit H3 (Abcam, ab219407, 1:1000), anti‐CD63 (Abcam, ab271286, 1:200), anti‐MPO (Abcam, ab208670, 1:100), anti‐NE (Abcam, ab131260, 1:250), and anti‐laminB1 (Abcam, ab16048; 1:1000), anti‐CD44 (Abcam, ab6124, 1:200), anti‐pERM (CST; 1:500), DAPI (Solarbio, D6470) and phalloidin (Abcam, ab176759). The cell membrane was stained with a PKH26 Red Fluorescent Cell Linker Mini Kit (Sigma, MINI26) according to the provided protocol before the cells were fixed. After staining, the cells were observed using a Zeiss LSM 900 confocal microscope.

Live cells were placed in a small incubator for visualization and fixed on the microscope stage. The incubator provided a constant temperature of 37°C and an atmosphere of 5% CO_2_.

### 2.12 Flow cytometry

Neutrophils purified from septic patients and healthy donors were stained with anti‐CD63 (PE, BioLegend, 353010), anti‐CD35 (FITC, BD, 565330), TUNEL (V450, BD, 561425), Annexin V PE/7‐AAD (BD, 559763) and CM‐H2DCFH‐DA (FITC, Solarbio, D6470). The samples were analysed by flow cytometry (FACSCanto II, BD Bioscience). Neutrophils were preincubated with or without cytochalasin B (CytB) before stimulation with PMA and then stained with anti‐CD63 (PE, BioLegend, 353010), anti‐CD35 (FITC, BD, 565330), Annexin V PE/7‐AAD (BD, 559763) and CM‐H2DCFH‐DA (FITC, Solarbio, D6470). The samples were analysed by flow cytometry (FACSCanto II, BD) and anti‐CD44 (PE, CST, 8724).

### 2.13 ELISA

Neutrophil nuclear proteins and cytoplasmic proteins were extracted, and the culture supernatants were obtained from neutrophils after stimulation with PMA. The procedure was carried out based on a kit protocol (RD, DY9167‐05, DY3174; Abcam, ab45912). Then, 100‐µl volumes of standards or samples were added to a 96‐well plate and incubated at RT. Samples were added to three wells each. Biotinylated antibody, streptavidin‐HRP reagent and TMB substrate were added sequentially according to the protocol. The colour was developed by incubation for 5–30 min at RT while the sample was protected from light. Stop solution was added to each well. The absorbance at 450 nm was measured within 5 min to measure protein levels, and the data were recorded.

### 2.14 Immunohistochemistry

Twenty‐four hours after CLP or sham operation, the lungs were taken from the mice, fixed in 4% formaldehyde, paraffin‐embedded and sectioned at 5 μm. The sections were exposed to 3% hydrogen peroxide for 10 min to inhibit endogenous peroxidase activity, followed by blocking with 3% bovine serum albumin for 30 min. The tissue sections were stained with haematoxylin and eosin (H&E) and then observed by light microscopy.

Tissue sections were prepared as described above and incubated with rabbit anti‐mouse Ly6G antibody (Abcam, ab238132, 1:2000) at 4°C overnight. The sections were then washed and incubated with anti‐rabbit IgG secondary antibody for 30 min at RT. Then, the sections were restained with haematoxylin and developed using a diaminobenzidine (DAB) kit. The results were obtained by examination under an IX73 microscope (Olympus).

Tissue sections were prepared as previously described.^19^ The sections were then incubated overnight with rabbit anti‐cit H3 antibody (Abcam, ab219407, 1:100) and rabbit anti‐MPO polyclonal antibody (Abcam, ab208670, 1:100) or rabbit anti‐cit H3 antibody and rabbit anti‐NE polyclonal antibody (Abcam, ab68672, 1:100). After washing, the slides were incubated with fluorescent dye‐coupled secondary antibodies for 1 h at RT. The DNA was stained with DAPI (Servicebio, G1012, 1:200) for 10 min. Images were acquired using a fluorescence microscope.

### 2.15 Statistical analyses

All statistical analyses were performed, and graphs were prepared with GraphPad Prism 8.0 software and Adobe Illustrator. The Shapiro–Wilk test was used to test the normality of continuous variables. The results are expressed as the mean ± standard deviation (SD). For group comparisons, one‐way ANOVA was used to compare continuous variables with a normal distribution. The Kruskal–Wallis test was used to compare continuous variables with a skewed distribution. Tukey's post hoc test or Dunn's post hoc test was used for multiple comparisons. Student's *t*‐test and the Wilcoxon paired signed rank test were used to compare differences between two groups. The Pearson correlation coefficient was used for correlation analysis. Significant differences are noted by asterisks (*p < 0.05, **p < 0.01, ***p < 0.001, ****p < 0.0001).

## 3 RESULTS

### 3.1 Sepsis leads to increased NET production and organ damage

**FIGURE 1:**
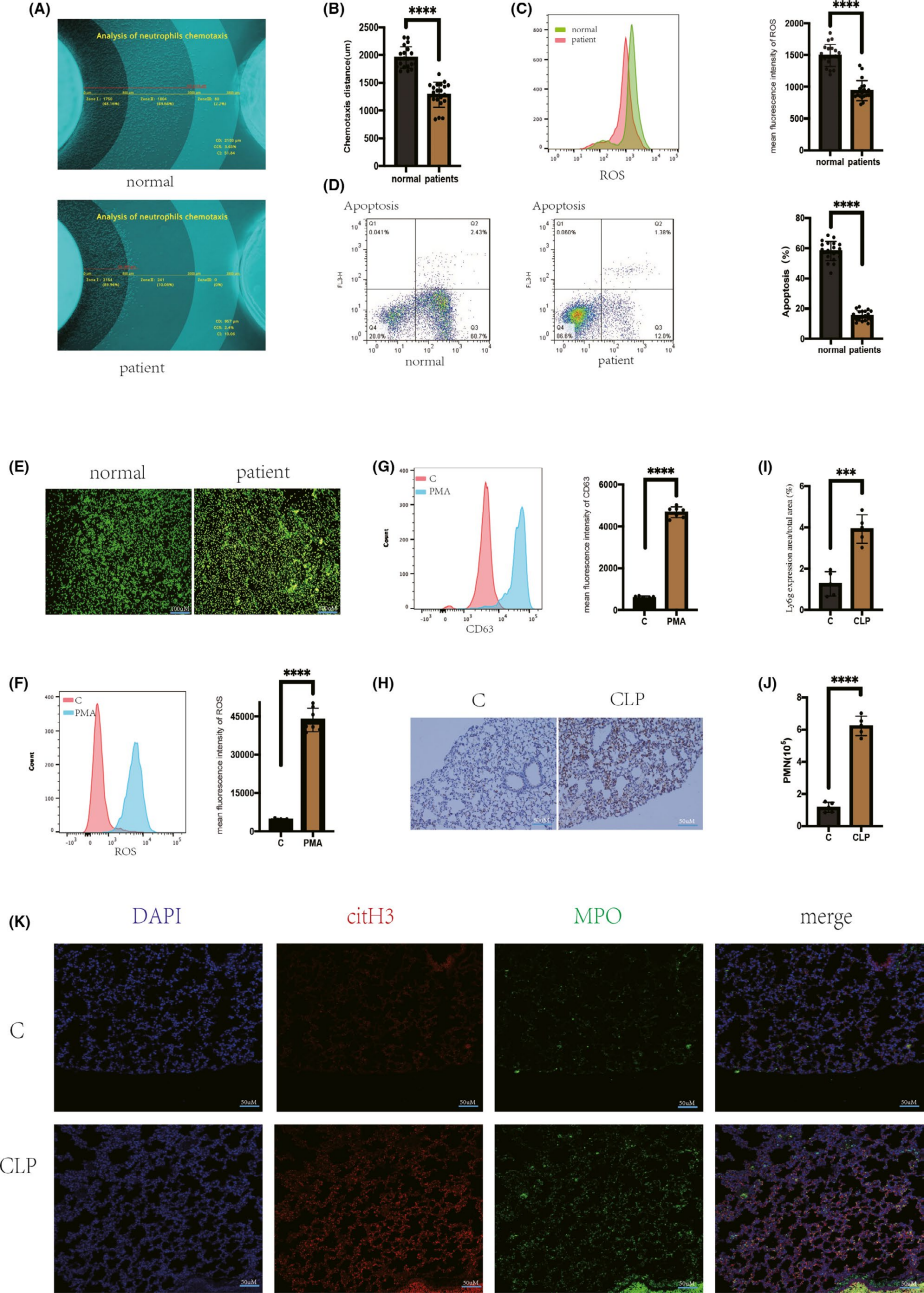
Neutrophil dysfunction and organ damage due to sepsis. (A) Agarose chemotaxis model to measure neutrophil chemotaxis distance, chemotaxis index. (B) Chemotaxis distance of sepsis patients was significantly smaller than that of normal controls (*p*<0.05). (C) PMA stimulation of neutrophils in sepsis patients and normal subjects, respectively, and flow cytometry detection of intracellular ROS formation. The ability of neutrophils to produce ROS was significantly lower in septic patients than in normal subjects (*p*<0.05). (D) Neutrophils from normal and septic patients were extracted separately and cultured in vitro for 24 h, and the apoptosis rate was detected by flow cytometry. Apoptosis of neutrophils was significantly delayed in sepsis patients compared with normal subjects. (E) Plasma from septic patients and normal subjects were isolated and stimulated separately with normal human neutrophils, and sytox neutrophil DNA staining (scale bar: 100 μm). Sepsis model of CLP mice (n=5) was established. (F) PMA (100 nM) stimulated neutrophils for 30 min, and ROS production was detected by flow cytometry. (G) PMA (100 M) stimulated neutrophils for 30 min, and cell membrane CD63 expression was detected by flow cytometry. (H) Ly6g‐positive cells in lung sections from both groups were detected by immunohistochemistry (scale bar: 50 μm). (I) The infiltration of neutrophils (ly6g +) in the lungs of septic mice was increased compared with the normal mice (*p* < 0.05). (J) In alveolar lavage fluid, CLP mice had increased neutrophils compared with normal mice (*p* < 0.05). K: Immunofluorescence was performed for citrullinated histones and MPO citrullinated histones

The functions of neutrophils collected from 20 septic patients and 20 healthy volunteers were investigated. Based on the modified chemotactic model designed by our group,^20^ we found that chemotaxis was significantly decreased in the neutrophils of septic patients (Figure 1A,B). However, these neutrophils exhibited significantly higher expression of an azurophil granule marker (CD63) and a secretory vesicle marker (CD35) than neutrophils from healthy volunteers (Figure S1A). In addition, PMA was used to stimulate the two groups of neutrophils, and the level of ROS produced by neutrophils from septic patients was significantly decreased (Figure 1C). Neutrophil apoptosis was markedly delayed in septic patients compared with healthy volunteers (Figure 1D). We found significantly higher plasma levels of histones in patients with sepsis than in normal subjects (Figure S1H). Stimulating normal neutrophils with septic patient‐derived plasma increased the production of NETs (Figure 1E,S1B). In contrast, the ability of neutrophils to produce NETs did not significantly differ between normal and septic patient‐derived cells stimulated with healthy patient plasma (Figure S1C). Neutrophils were stimulated with PMA, and NET‐related functions, including ROS production and surface CD63 levels, were examined (Figure 1F,G). TUNEL staining showed significantly higher DNA breaks in the nuclei of intact neutrophils in patients than in healthy individuals (Figure S1D). In addition, TUNEL staining of neutrophils was significantly higher in patients than in healthy individuals (Figure S1E,F). We found significantly higher levels of cit H3 in the nuclei of neutrophils from patients than in those from healthy subjects (Figure S1G).

We established a model of sepsis in CLP mice for 24 h. Alveolar destruction with massive cellular infiltration was observed in the lungs (Figure S2B). Several internal organs, including the kidneys, spleen and liver, were congested and oedematous (Figure S2A). Lung tissue was immunostained using anti‐ly6G, and mice in the CLP group exhibited significantly increased perialveolar neutrophil infiltration (Figure 1H,I). Furthermore, neutrophils in alveolar lavage fluid in the CLP group were significantly increased (Figure 1J). In addition, the levels of multiple inflammatory cytokines in alveolar lavage fluid were increased in the CLP group (Figure S2C). Fluorescent staining revealed a large amount of citrullinated histones carrying MPO and NE in the lung tissue of CLP mice (Figure 1K, S2D). Furthermore, TUNEL staining confirmed increased apoptosis in lung tissues in the CLP group (Figure S2E).

### 3.2 MPO that is free outside the granule cannot enter the nucleus directly

**FIGURE 2:**
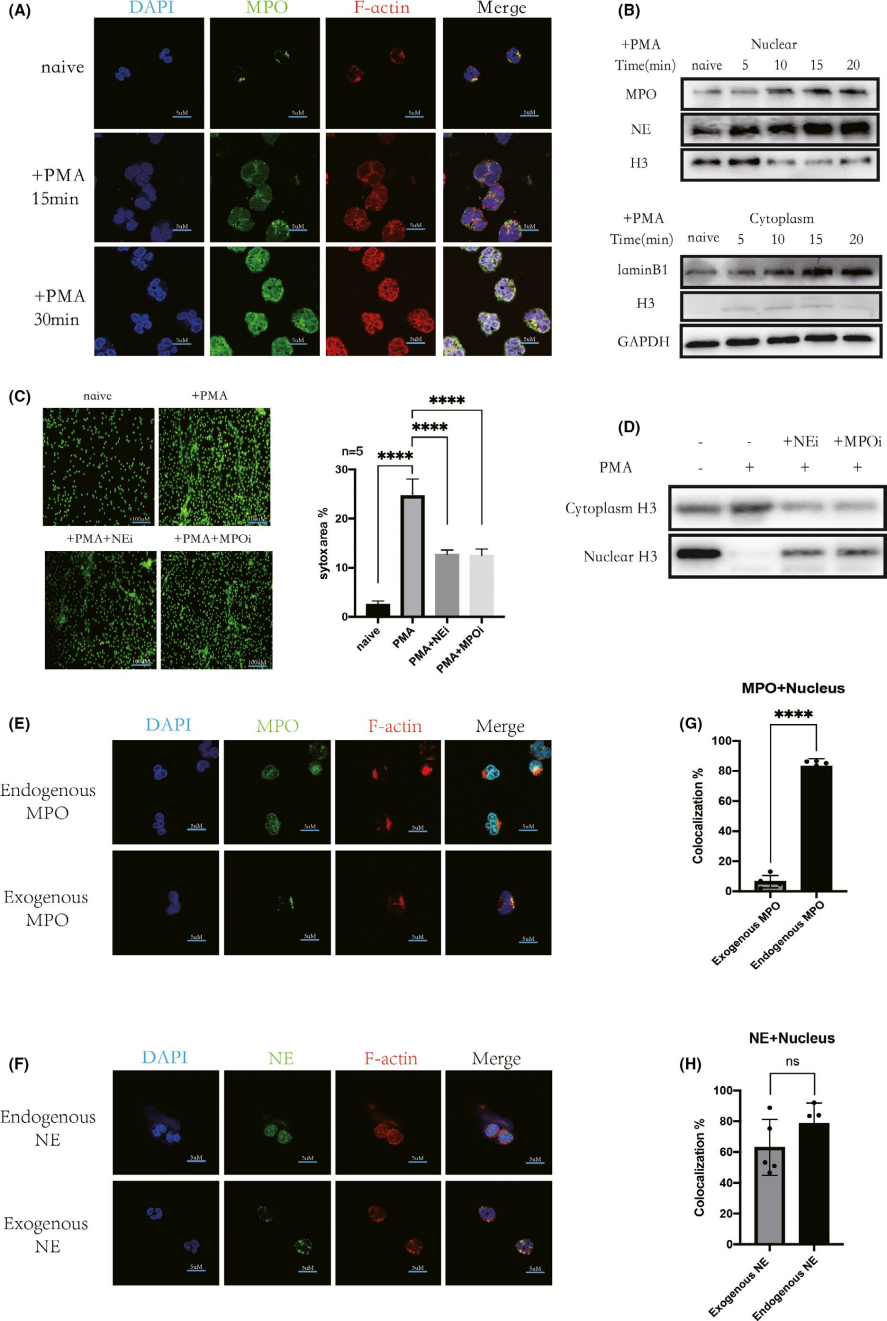
MPO that is free outside the particle cannot enter the nucleus directly. (A) Fluorescent staining of MPO, F‐actin, nuclei in normal neutrophil after PMA (100 nM) stimulation (15 min, 30 min), respectively, with a gradual increase in MPO (green) co‐localization with the nucleus (blue) (scale bar: 5 μm). (B) After PMA (100 nM, 0–20 min) activation of neutrophils, WB detected an increase in intranuclear MPO, NE, and a decrease in histone (H3) content over time. In contrast, the cytoplasmic inner nuclear membrane component (LaminB1) and histone component gradually increased with time. (C) Neutrophils were preincubated with NE activity inhibitor (NEi, 2 μM) and MPO activity inhibitor (MPOi, 1 μM) for 30 min, and then stimulated with PMA (100 nM, 120 min), NET could still be produced. Separately use sytox (green) for DNA staining (scale bar: 100 μm). However, compared with PMA stimulation alone, the amount of NETs produced by neutrophils decreased significantly. (D) Using WB to detect histones in the nucleus and cytoplasm, both NEi and MPOi can reduce the degradation of histones in the nucleus compared to PMA (100 nM, 30 min) stimulation alone. (E and F) Fluorescent staining of exogenous MPO and NE, respectively, followed by incubation with neutrophils for 30 min, and stimulation of neutrophils with PMA (100 nM) for 30 min, compared with direct fluorescent staining of intracellular MPO and NE. (E and G) co‐localization of endogenous MPO (G, green) with the nucleus (G, blue) increased, while exogenous MPO co‐localized less with the nucleus(*p* < 0.05) (scale bar: 5 μm). (F and H) endogenous NE and exogenous NE co‐localized with the nucleus were both increased and not significantly different (*p* >0.05) (scale bar: 5 μm)

Within 30 min of PMA stimulation, neutrophils produced small amounts of NETs, while most cells remained intact (Figure S3A,B). DNA breaks were present in the nuclei of morphologically intact neutrophils (Figure S3C). We investigated these early intranuclear changes in NET generation within 30 min of PMA stimulation and found similarities to patient neutrophils (Figure S1F). A large amount of MPO and NE was carried by NETs.^5^, ^21^ PMA stimulation increased the levels of nuclear MPO and NE over time (Figure 2A,B, S4A). Interestingly, we also found large amounts of MPO in the nuclei of patient‐derived neutrophils (Figure S3D). Additionally, the increase in MPO translocation to the nucleus occurred later than the increase in extracellular MPO (Figure S4A).

Histones were decreased with increasing levels of MPO and NE in the nucleus, and histones appeared in the cytoplasm (Figure 2B). Additionally, the level of the nuclear membrane component LaminB1 was increased in the cytoplasm (Figure 2B). We also observed the loss of the nucleus early after stimulation with PMA but did not observe this effect in normal neutrophils (Movie 1), and DNA was eventually released to form NETs (Movies 2–4). To further verify the involvement of MPO and NE in histone degradation, we used enzyme inhibitors. NET production decreased significantly after the addition of enzyme inhibitors (Figure 2C). Both inhibitors inhibited the degradation of histones in the nucleus (Figure 2D). Interestingly, the two inhibitors seemed to utilize different mechanisms of action. MPOi reduced the entry of NE into the nucleus (Figure S4B) but did not significantly reduce the entry of MPO into the nucleus (Figure S4B). In contrast, NEi did not reduce the entry of either MPO or NE into the nucleus (Figure S4B). After stimulation with PMA for 30 min, the co‐localization of azurophil granules with MPO and NE was significantly decreased (Figure S4C,E,F). However, most MPO and NE had been transferred into the nucleus (Figure S4C–E).

Exogenous MPO and NE were first stained and then incubated with neutrophils for 30 min. Exogenous MPO was unable to enter the nucleus after neutrophils were stimulated with PMA for 30 min (Figure 2E,G). In contrast, exogenous NE could enter the nucleus directly (Figure 2F,H).

### 3.3 Azurophil granules fuse with the nuclear membrane and degranulate to the nucleus

**FIGURE 3:**
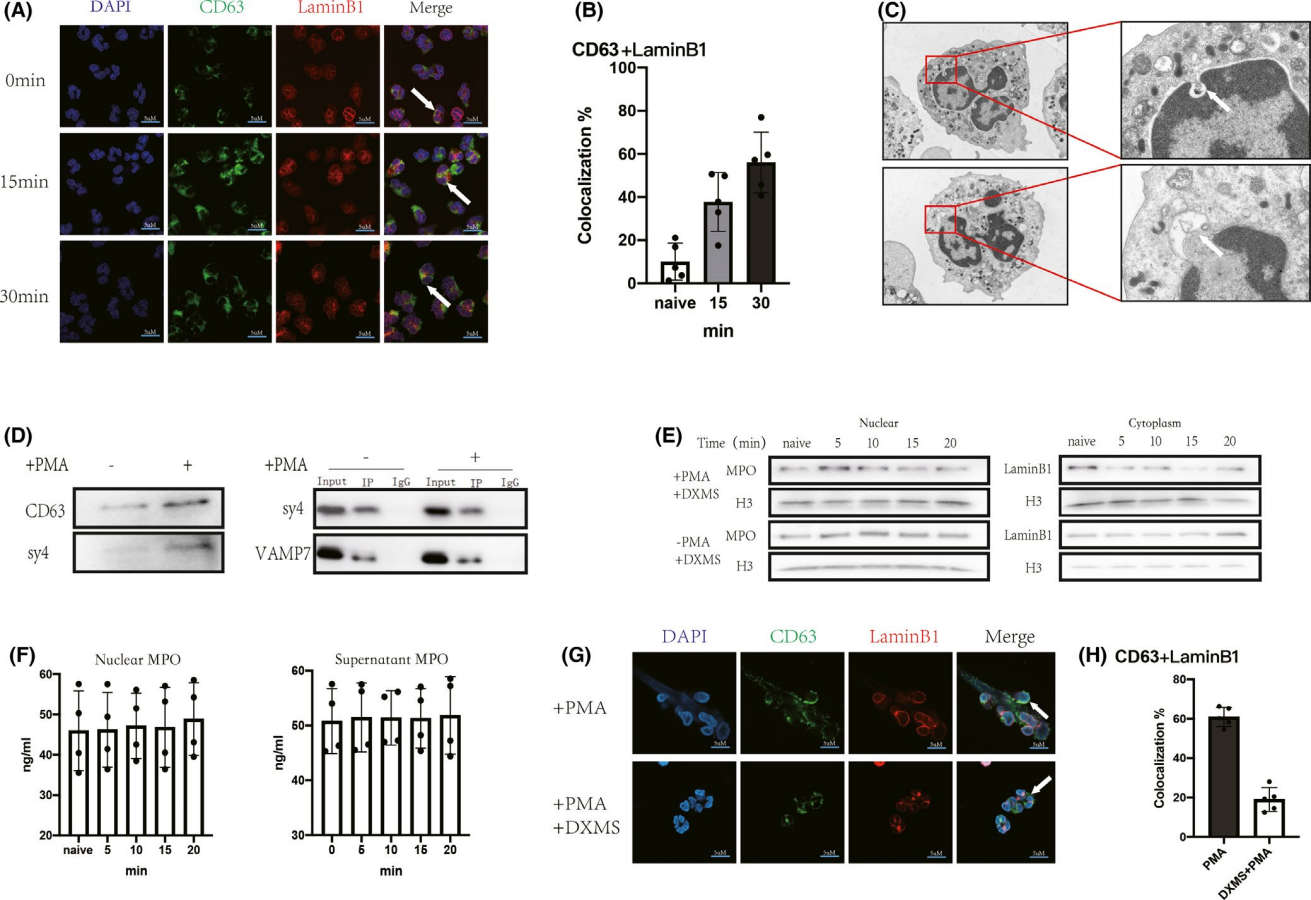
Azurophil granules fuse with nuclear membrane and degranulate to the nucleus. (A and G) Fluorescent staining of CD63, nuclear membrane (LminB1) and nucleus (DAPI), respectively. (A) Gradual increase in co‐localization of CD63 (green) with LaminB1 after PMA (100 nM, 15, 30 min) stimulation of neutrophils (scale bar: 5 μm) (↑: Gradual increase in co‐localization of CD63 with the nuclear membrane). (B) image J analysis of gradual increase in co‐localization of CD63 with nuclear membrane. (C) Electron microscopic observation of neutrophils after PMA (100 nM, 30 min) stimulation of neutrophils. The nuclear membrane of neutrophils fused with the granule membrane and endocytosed the endoparticle components. (D) Nuclear membrane proteins were extracted from normal neutrophils and neutrophils after PMA (100 nM) stimulation for 30 min, respectively. WB measured CD63 and Syntaxin4 (sy4) expression in the nuclear membrane increased after PMA stimulation of neutrophils. Extraction of nuclear membrane proteins, co‐ip showing co‐localization of sy4 with vamp7. E‐H: Dexamethasone (100 µM) was coincubated in neutrophils for 30 min, followed by PMA stimulation of neutrophils. (E) WB measured the constant amount of MPO and histone in the nucleus, and there was no significant increase in the cytoplasmic inner LminB1 and histone. (F) ELISA measured the concentration of MPO in the nucleus, and the expression was constant. (G) PMA (100 nM, 30 min) stimulation with or without dexamethasone (100 µM, 30 min) pre‐incubation of neutrophils, the degree of CD63 co‐localization with nuclear membrane was observed using immunofluorescence (scale bar: 5 μm) (↑: Co‐localization of CD63 with the nuclear membrane). (H) image J analysis of CD63 co‐localization with nuclear membrane, dexamethasone conferred a significant inhibition of co‐localization

We hypothesized that targeted nuclear degranulation is responsible for the transfer of MPO into the nucleus. Fluorescent staining showed a gradual increase in the co‐localization of CD63 with nuclear membranes after PMA activation of neutrophils (Figure 3A,B). Scanning electron microscopy also showed particle fusion with the nuclear membrane (Figure 3C). After PMA stimulation, the expression of CD63 and sy4 on the nuclear membrane increased (Figure 3D), and co‐immunoprecipitation also showed increased binding of VAMP7 to sy4 on the surface of the nuclear membrane (Figure 3D).

Dexamethasone is a classic reagent that can inhibit degranulation^22^ and can stabilize membranes. Neutrophils were pretreated with dexamethasone and then stimulated with PMA. MPO in the nucleus was not increased over time (Figure 3E,F). The decrease in histones was abrogated (Figure 3E). Furthermore, no increase in MPO in the supernatant was observed (Figure 3F). Immunofluorescence analysis showed that CD63 co‐localization with the nuclear membrane was reduced (Figure 3G,H).

### 3.4 Targeted nuclear degranulation is dependent on changes in actin

Actin began to repolymerize at approximately 10–15 min after PMA‐induced activation in neutrophils (Movies 1 and 5) when DNA depolymerization occurred. In contrast, no significant change in F‐actin or nuclei was observed in control neutrophils (Movie 6). The PMA‐induced increase in MPO in the nucleus was effectively inhibited by 100 nM CytB, and the degree of histone destruction was significantly reduced (Figure S5A,B). PMA was used to stimulate CytB pretreated neutrophils. Cellular morphology remained intact, and no significant change in nuclear morphology was observed (Movie 7).

**FIGURE 4:**
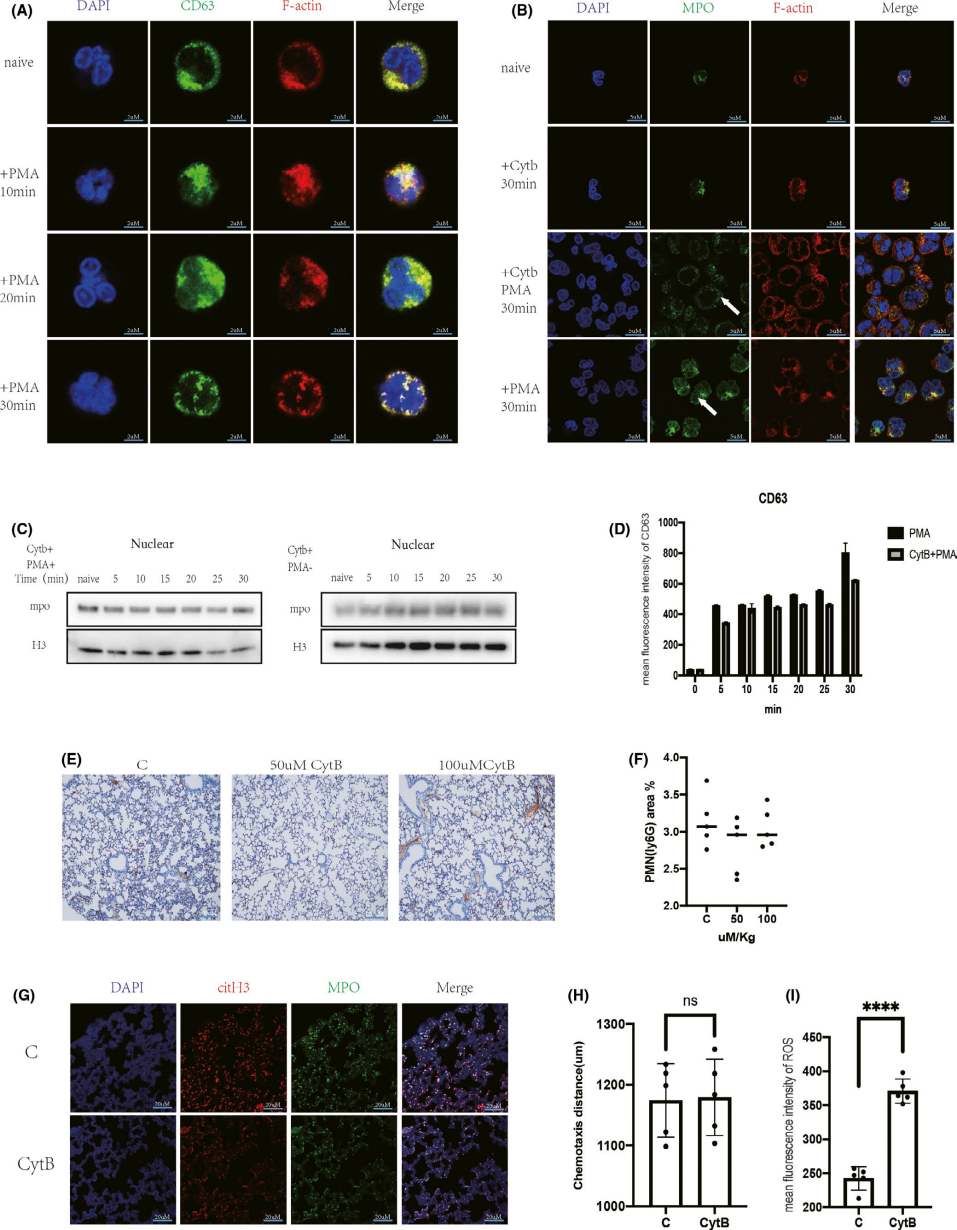
Degranulation to the nucleus is dependent on actin changes. (A and B) Fluorescent staining of neutrophils for CD63, MPO, F‐actin, cell membrane (PM) and nuclei, respectively (scale bar: 5 μm). (A) PMA (100 nM, 0–30 min) stimulation of neutrophils resulted in synchronous changes in granules (CD63, green) and F‐actin (red). (B) Incubation of neutrophils with cytochalasin B (CytB, 100 nM) did not increase the expression of neutrophil MPO but reduced co‐localization of MPO with the nucleus after PMA (100 nM, 0–30 min) stimulation (↑: CytB inhibits the entry of PMA‐stimulated neutrophil MPO into the nucleus). (C and D) CytB (100 nM) pre‐incubation of neutrophils after PMA (100 nM, 0–30 min) stimulation of neutrophils. (C) WB showed no significant increase in nuclear MPO content over time, and histone content was constant in the nucleus. (D) Flow cytometry detected CD63 expression on the cell membrane surface of neutrophils, and CytB (100 nM) did not affect degranulation. (E and G) CLP mice were modelled for 24 h, and lung tissue was taken. (E) Lungs were injected intraperitoneally with 50 and 100 µM/kg before modelling. Lungs were immunohistochemically stained for Ly6G. (F) Image j software Analysis of lung neutrophils (Ly6G). (G) Lungs were labelled with citH3 (red), MPO (green) and nuclei (DAPI) using immunofluorescence. H‐I: Peripheral blood was taken from CLP mice. (H) Agarose gel model to detect neutrophil chemotaxis. (I) Flow cytometry to detect neutrophil ROS production capacity after PMA stimulation of neutrophils

Fluorescent staining showed the co‐localization of CD63 with F‐actin after PMA stimulation. (Figure 4A). PMA stimulation of cytB‐preincubated neutrophils did not affect the increase in azurophil granules containing MPO (Figure 4B), but the amount of MPO that entered the nucleus was clearly reduced (Figure 4B,C, S5D). CytB inhibited the PMA‐induced alterations in CD63 distribution (Figure S5C). Interestingly, azurophilic granule degranulation appeared to have less of an effect than targeted nuclear degranulation (Figure S4A, S5D). Flow cytometric analysis of neutrophil membrane CD63 expression showed no effect of CytB on PMA‐stimulated degranulation (Figure 4D).

We also tested the effects of CytB on neutrophil apoptosis (Figure S5E,F), oxygen burst capacity (Figure S5G,H) and chemotaxis (Figure S5I,G). Increasing concentrations of CytB affected centriole degranulation (Figure S6A,B), chemotactic function (Figure S6C) and oxygen burst capacity (Figure S6D).

CLP mice were intraperitoneally injected with CytB, and lung tissue was collected 24 h later. CytB effectively reduced pulmonary oedema in CLP mice at concentrations of 50 µM/kg and 100 µM/kg (Figure 4E), but the proportion of neutrophil infiltration was not reduced (Figure 4F). Immunofluorescence analysis showed that CytB reduced the formation of NETs (citH3+MPO) in the lung (Figure 4G). CytB reduced broken DNA in mouse lungs, as shown by TUNEL staining (Figure S6E,F). Neutrophils were isolated from the peripheral blood of CLP mice 24 h after CytB treatment. CytB had no effect on peripheral blood neutrophil chemotaxis in CLP mice (Figure 4H, S6G) but increased ROS production after PMA stimulation (Figure 4I, S6H).

### 3.5 CD44 translocation to the nucleus and delayed targeted nuclear degranulation are mediated by CD44/ERM/F‐actin

**FIGURE 5:**
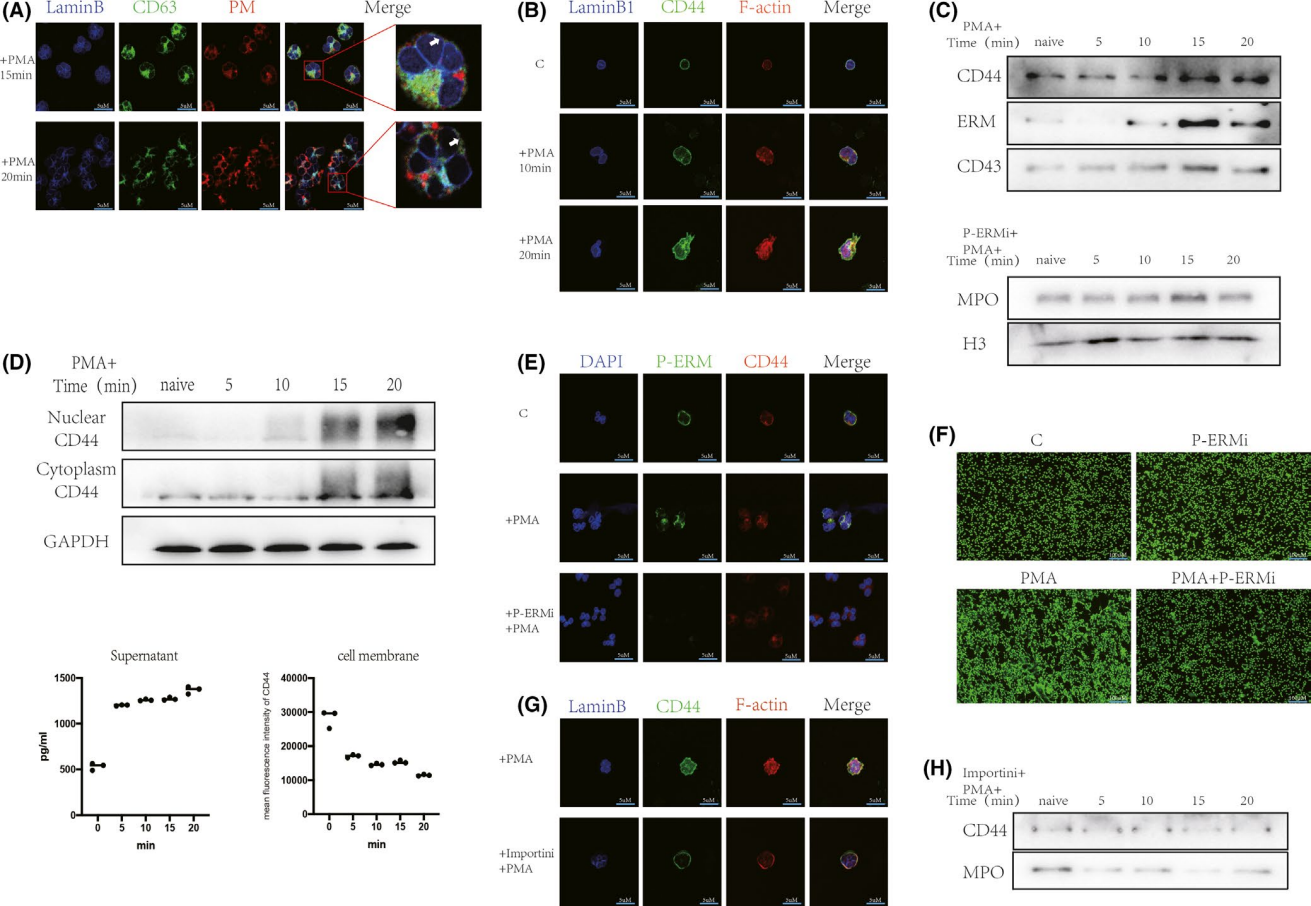
CD44 translocation to the nucleus and delayed ‘targeted nuclear degranulation’ mediated by CD44/ERM/F‐actin. (A) Neutrophils were fluorescently stained for nuclear membrane, cell membrane and CD63, respectively. After stimulation of neutrophils with PMA (100 nM), CD63 co‐localization with the cell membrane (15 min) was earlier than CD63 co‐localization with the nuclear membrane (20 min) (scale bar: 5 μm). (B) PMA (100 nM) stimulates neutrophils for 0–20 min. Immunofluorescence staining of nuclear membrane (LaminB1,blue), skeleton (F‐actin, red) and CD44 (green), respectively. (C) PMA (100 nM) stimulates neutrophils for 0–20 min. WB for changes in nuclear membrane CD44, ERM and CD43 content changes. ERM phosphorylation inhibitor (P‐ERMi, 10 µM, 30 min) pre‐incubation of neutrophils for 30 min. WB detection of intracellular MPO, histone (H3) changes. (D) WB and ELISA for changes in intranuclear, cytoplasmic and extracellular CD44 content, flow cytometry for changes in cytosolic CD44 content. (E and F) P‐ERMi (10 µM, 30 min) pre‐incubation of neutrophils. (E) PMA (100 nM) stimulates neutrophils for 30 min, immunofluorescence staining for nuclear (DAPI,blue), P‐ERM (green) and CD44 (red). (F) PMA (100 nM) stimulates neutrophils for 120 min, SYTOX fluorescence staining. (G) Importini pre‐incubation of neutrophils, immunofluorescence staining of nuclear membrane (LaminB1,blue), skeleton (F‐actin, red) and CD44 (green). (H) Importini (30 µM) pre‐incubation of neutrophils, WB detection of nuclear membrane CD44 and changes in nuclear MPO content

We performed fluorescence staining of the nuclear membrane and cell membrane. CD63 was expressed earlier in the cell membrane than in the nuclear membrane (Figure 5A). In the present study, CD44 localization in the cytoplasm and nucleus was increased by PMA stimulation (Figure 5B). Cytosolic CD44 decreased sharply after stimulation, while cytoplasmic, nuclear and extracellular CD44 expression increased (Figure 5D). Interestingly, we detected an abrupt increase in CD44, CD43 and ERM in the nuclear membrane approximately 15 min after stimulation (Figure 5C). We inhibited the F‐actin‐nuclear membrane complex using an ERM phosphorylation inhibitor (ERMi) and found that the increased nuclear MPO and histone degradation were inhibited and that ERM was not detectable in the nuclear membrane (Figure 5C). However, CD44 translocation to the nucleus was not affected (Figure 5E). Pre‐incubation of neutrophils with ERMi significantly inhibited NET formation (Figure 5F). An importin‐β inhibitor (Importini) inhibited the increase in CD44 at the nuclear membrane and the increase in MPO in the nucleus (Figure 5G). This treatment significantly inhibited the transfer of CD44 to the nucleus (Figure 5H).

## 4 DISCUSSION

Neutrophils are an important component of innate immunity and the immune cell that arrives first at the site of infection. Neutrophils eliminate pathogens by phagocytosis, degranulation and the release of ROS. Neutrophils also play a role in antigen presentation. However, based on investigations into neutrophil function, neutrophils have been shown to play dual roles during infection.^23^ Activated neutrophils release cytokines, ROS, proteases, MPO and other enzymes, which can lead to bystander tissue damage.^24^ We found that sepsis results in delayed neutrophil apoptosis and decreased chemotaxis. Therefore, although the number of neutrophils is increased in sepsis, neutrophils cannot reach the specific site of inflammation. We also found that sepsis results in the extensive activation of neutrophils and increased degranulation, which may be initial effects of exacerbated multiorgan dysfunction in septic patients.

Reduced neutrophil chemotaxis and bactericidal function and the extensive degranulation of neutrophils in vivo may be factors that exacerbate organ dysfunction. We also found that plasma from septic patients could stimulate the excessive release of NETs from neutrophils. Maruchi, Y. et al. reported that high levels of NET‐MPO in septic patients were associated with the severity of organ dysfunction. In addition, NET‐MPO levels could predict the prognosis of septic patients.^25^ NETs are strongly adherent and carry various hydrolytic enzymes, which further aggravates local tissue damage. Multiple organ damage in sepsis is associated with extensive neutrophil activation in vivo.^26^ Typically, some neutrophils are retained in perialveolar capillaries.^27^ In this study, we utilized a mouse model of sepsis to verify the damage caused by neutrophils. Since capillary diameter in the lung is smaller than the size of neutrophils, many neutrophils accumulate in the lung during infection.^27^ We confirmed these points through histochemical analysis. We found a large amount of accumulated citrullinated histones in the lungs of septic mice, and large amounts of MPO and NE were carried on the surfaces of histones. Furthermore, apoptosis in the lungs was increased. This finding further illustrates that NETs carrying enzymes are one of the important causes of organ dysfunction. NETs cause neutrophil death that differs from apoptosis and necrosis and is thought to involve the active release of DNA strands from neutrophils. DNA unwinding and fragmentation are important steps in the formation of NETs.^28^ Although additional upstream pathways are involved in the release of NETs from activated neutrophils, the degradation of histones in the nucleus is thought to be an essential pathway for DNA unwinding.^7^, ^29^, ^30^ PMA is commonly used to promote the production of NETs in neutrophils.^31^ MPO, NE and PAD4 are the primary hydrolases that promote nuclear histone depolymerization. Several studies have also demonstrated that NE can enter the cytoplasm from granules and then translocate to the nucleus.^6^, ^32^ However, no studies have shown how MPO translocates into the nucleus. We also demonstrated that MPO and NE are transferred to the nucleus when NETs are formed. In addition, the formation of NETs could be reduced using active inhibitors of both MPO and NE. Interestingly, these inhibitors seemed to use different mechanisms of action. MPOi reduced the entry of NE but not MPO into the nucleus. Metzler, K.D. et al. showed that ROS trigger NE translocation from granules into the cytoplasm, activating the proteolytic activity of NE in an MPO‐dependent manner, after which NE is ultimately transferred to the nucleus.^8^ It has also been noted that MPO alone does not increase the degradation of histones.^6^ Therefore, MPO may reduce the formation of NETs by reducing the transfer of NE to the nucleus. NEi did not reduce the translocation of MPO or NE into the nucleus, which may be because the enzymatic activity of NE is necessary for histone degradation. However, the process by which these enzymes are transferred into the nucleus is still unclear. Some studies have suggested that, in addition to NE, PAD4 can be transferred to the nucleus through the nuclear pore. It has also been suggested that GSDMD pores in the nuclear and granule membranes form, which can mediate the transfer of enzymes.^10^ We confirmed in our study that after neutrophil activation, some NE and MPO were released outside granules, and their co‐localization with the nucleus was increased. However, the diameters of the nuclear pores and GSDMD pores are small, and neither pore is sufficient to allow the passage of large molecules, such as MPO. We found that exogenous MPO, which is free in the cytoplasm, cannot enter the nucleus, whereas NE can. Therefore, we believe that MPO cannot enter the nucleus through pores in the nuclear membrane. Neutrophil degranulation refers to fusion of the granular membrane and cell membrane and the release of intragranular material via exocytosis. We suggest that degranulation to the nucleus occurs. Azurophil granules move towards the nuclear membrane and fuse, releasing granule contents and transferring MPO to the nucleus. In the present study, we found that the stimulation of neutrophils with PMA for 30 min resulted in DNA breakage in the nucleus. Based on this finding, we concluded that within 30 min, MPO entered the nucleus, and this effect was confirmed by an assay to detect nuclear proteins. We then measured nuclear membrane proteins and found that CD63 was present, which indicated that the nuclear membrane had fused with the azurophil granular membrane. Moreover, increased sy4 expression in the nuclear membrane was found, and this protein facilitates mutual recognition for fusion of the nuclear and granular membranes. Dexamethasone was effective in inhibiting neutrophil degranulation^33^ and had a membrane‐stabilizing effect. Dexamethasone also inhibited fusion of the nuclear membrane with granules, and the increase in MPO in the nucleus was also inhibited. This finding also demonstrated that MPO entered the nucleus through the targeted nuclear degranulation pathway. In our study, we found strong overlap between the localization of azurophilic granules and F‐actin in neutrophils after their activation. Most studies suggest that the movement of azurophilic granules is associated with F‐actin.^34^, ^35^

NETosis requires actin rearrangement, and F‐actin becomes more prominent during the first 30 min after PMA stimulation.^36^ Although studies have shown that cytochalasin effectively inhibits NETs, the exact mechanism is not known.^36^ In our study, we found that the nucleus underwent deagglutination after F‐actin repolymerization, which may have been caused by the crawling of azurophilic granules on the surface of F‐actin towards the nuclear membrane, which is the final step of targeted nuclear degranulation.^37^ We then used CytB to inhibit the repolymerization of F‐actin and found that azurophil granules were evenly distributed in the cytoplasm and that the co‐localization of MPO and the nucleus was significantly reduced. Interestingly, at the dose we used, CytB could inhibit the production of NETs but did not seem to have an effect on degranulation. Trendowski M et al. reported that intraperitoneal injection of cytochalasin b increased the effect of chemotherapy in leukaemic mice and had reduced cytotoxicity.^38^ We further administered CytB to a CLP model and found that CytB reduced pulmonary oedema during sepsis and did not affect the proportion of neutrophil infiltration. Small doses of CytB were effective in reducing the formation of NETs in the lungs of septic mice and did not affect neutrophil chemotaxis or enhance ROS production in peripheral blood. We therefore concluded that appropriate doses of CytB in sepsis could reduce inflammation by attenuating the formation of NETs and did not affect neutrophil chemotaxis to the site of inflammation.

We also found that degranulation to the outside of the cell seemed to occur earlier than targeted nuclear degranulation. CD44/ERM‐mediated binding of F‐actin to the plasma membrane is fundamental for the maintenance of neutrophil morphology, migration and degranulation. A. Bartolazzi et al. showed that PMA induced CD44 cleavage and was associated with MMPs.^39^ In our study, we also found a dramatic decrease in the cell membrane expression of CD44 after PMA stimulation, which provides a theoretical basis for the shedding and degradation of neutrophil cortical proteins. However, there was an increase in extracellular and intracellular CD44 expression, which may be caused by shedding of cell membrane CD44. The transfer of CD44 to the nucleus is considered to be one of the important mechanisms of metastasis in many diseases, including tumours,^17^ and the partial CD44 ICD or even full‐length CD44 can act as a signal to transfer to the nucleus and regulate gene expression. In our study, we also confirmed CD44 transfer to the nucleus in neutrophils. The expression of CD44 and ERM was detected in the nuclear membrane, providing a basis for the complex of F‐actin with the nuclear membrane. The linker of the nucleoskeleton and cytoskeleton (LINC) spans the double nuclear membrane and mediates nuclear‐cytoskeletal coupling.^40^ However, LINC expression is downregulated in mature neutrophil nuclei.^41^ Thus, the CD44/ERM/f‐actin complex may be the primary mechanism of nuclear‐cytoskeletal coupling in mature neutrophils. In our study, we found that the expression of CD44 in the nuclear membrane increased approximately 15 min after PMA stimulation, which might be the reason that targeted nuclear degranulation occurred later than degranulation. Inhibiting CD44 translocation to the nucleus and ERM phosphorylation could effectively inhibit targeted nuclear degranulation and NET formation. The delayed targeted nuclear degranulation of neutrophils under strong stimulation provided sufficient time for rapid degranulation to the extracellular compartment, avoiding the rapid formation of NETs by neutrophils under strong stimulation and the disintegration of cell membranes, resulting in the irregular release of large amounts of intracellular material.

**FIGURE 6:**
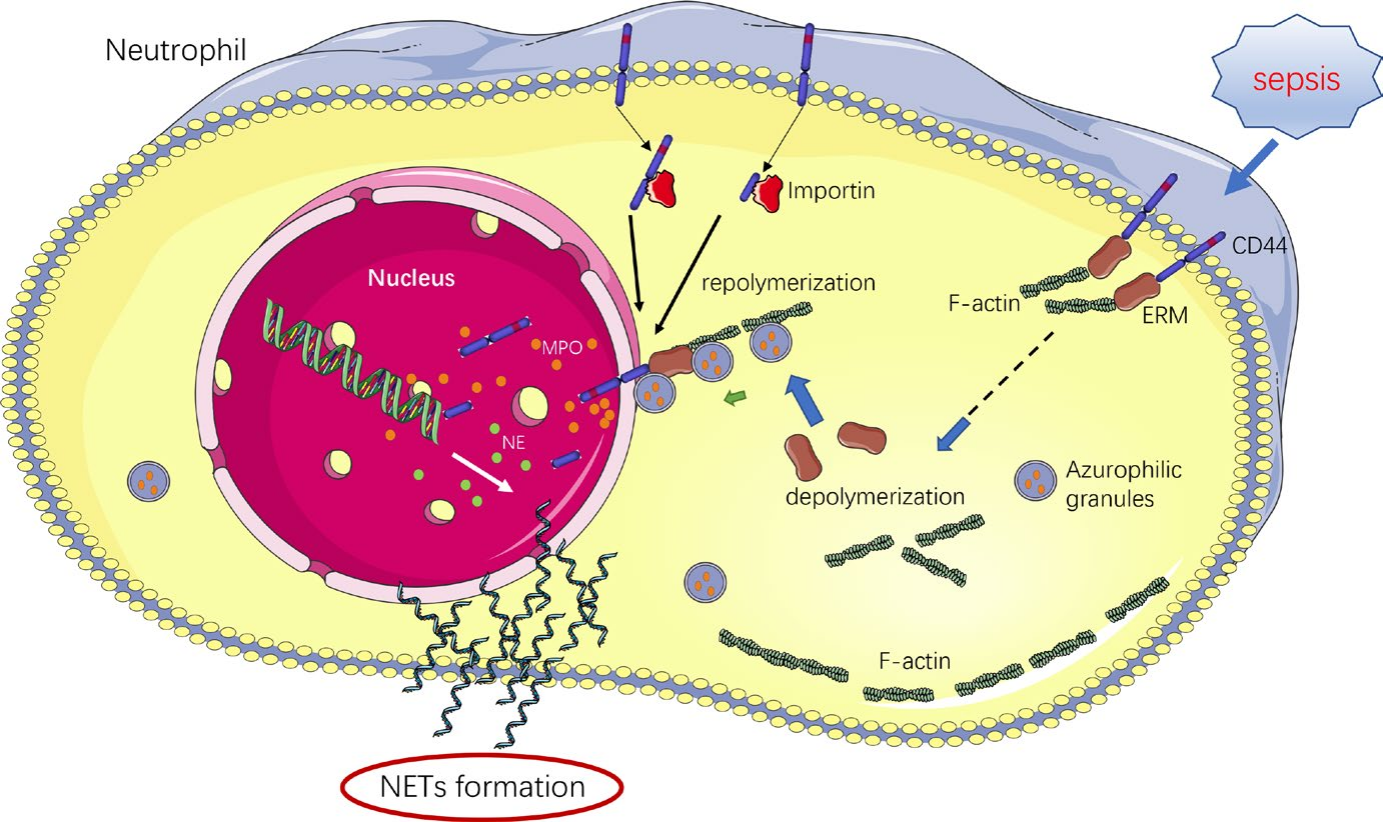
Schematic illustration of the mechanism of targeted nuclear degranulation. Upon activation of neutrophils, some CD44 is shed intracellularly and transported to the nucleus and nuclear membrane with the assistance of importin. Full‐length CD44 binds to the ERM at the nuclear membrane and further attaches to the recombinant F‐ actin, anchoring the F‐actin to the nuclear membrane. Tensinophilic granules extend the movement of F‐actin towards the nucleus and fuse with the nuclear membrane, releasing MPO and other enzymes into the nucleus. The large amount of MPO in the nucleus assists in the depolymerization and release of histone DNA

In summary, our research has revealed a new biological behaviour of neutrophils known as targeted nuclear degranulation (Figure 6). We have also elucidated the pathway by which MPO enters the nucleus during the formation of NETs. This study provides a new therapeutic target for multiple organ dysfunction caused by the excessive formation of NETs in sepsis. We have also elucidated the mechanism of delayed degranulation to the nucleus. Additionally, because a large number of enzymes and active factors are contained within neutrophil granules, targeted nuclear degranulation provides many possibilities for altering the nuclei of neutrophils. The findings of this study provide new insight for examining the behaviour and function of neutrophils.

## CONFLICTS OF INTEREST

The authors declare that they have no competing interests.

## AUTHOR CONTRIBUTIONS

YS, LL and BS designed the study and wrote paper. LL, YY, JH and DJ performed experiments. YZ, YC and ZZ performed collection of clinical data. YS and BS performed the statistical analysis. All authors read and approved the final manuscript.

## AUTHOR CONTRIBUTION

**Bingwei Sun:** Conceptualization (equal); Writing – original draft (equal). **yiming Shao:** Conceptualization (equal); Data curation (equal); Formal analysis (equal); Writing – original draft (equal). **lu Liu:** Conceptualization (equal); Data curation (equal). **linbin Li:** Data curation (equal); Funding acquisition (equal). **yunxi Yang:** Funding acquisition (equal); Investigation (equal). **jiamin Huang:** Methodology (equal); Project administration (equal). **dongdong Ji:** Project administration (equal); Resources (equal). **yuying Zhou:** Software (equal); Validation (equal). **yi Chen:** Investigation (equal); Visualization (equal). **zhechen Zhu:** Methodology (equal); Visualization (equal).

## Supporting information

Supplementary Material

Video S1

Video S2

Video S3

Video S4

Video S5

Video S6

Video S7

## Data Availability

Data are available from the authors upon reasonable request.
