# Supplementary material for: CD44/ERM/F‐actin complex mediates targeted nuclear degranulation and excessive neutrophil extracellular trap formation during sepsis
Source: J Cell Mol Med. 2022 Feb 11;26(7):2089–103. doi: 10.1111/jcmm.17231 (PMC8980940; doi:10.1111/jcmm.17231)
Supplement: Supplementary file 1 — Supplementary Material [file JCMM-26-2089-s001.docx]

**Supporting Information for**

**CD44/ERM/F-actin complex mediates targeted nuclear degranulation and excessive neutrophil extracellular trap formation during sepsis**

Yiming Shao, Linbin Li, Lu Liu, Yunxi Yang, Jiamin Huang, Dongdong Ji, Yuying Zhou, Yi Chen, Zhechen Zhu, Bingwei Sun**^*^**

**^*^** **Corresponding author**

Email: [sunbinwe@hotmail.com](mailto:sunbinwe@hotmail.com)

**This file includes:**

Figures S1 to S6

Movie S1 to S7

**
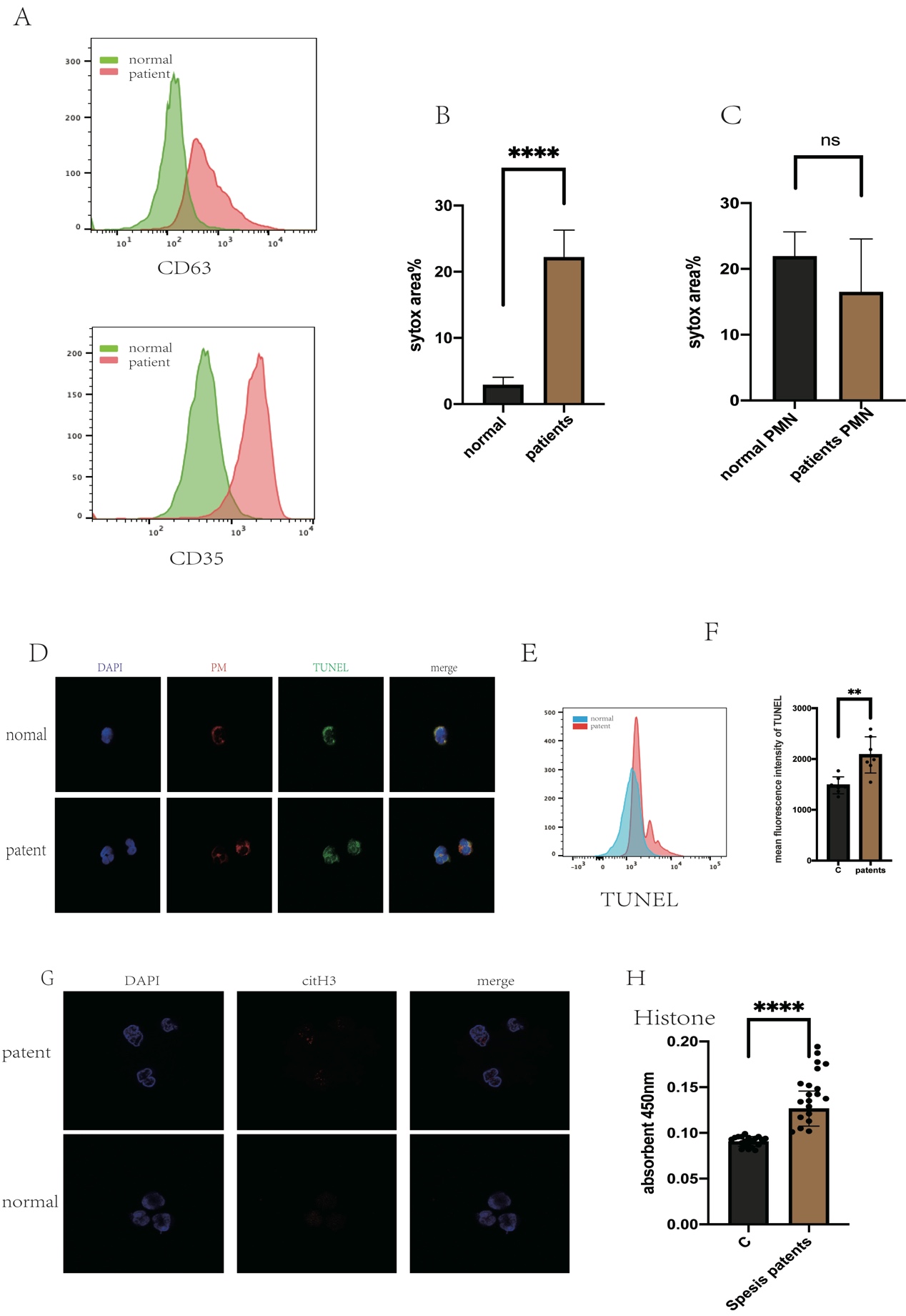
**

**Figure S1. Histone depolymerization and DNA breakage in the nucleus of neutrophils from patients with sepsis.** A: Flow cytometry detection of CD63 and CD35 on the surface of neutrophils was significantly higher in sepsis patients than in normal subjects. B: Analysis of the area of the extracellular DNA (NETs) region using image J. Plasma from septic patients stimulated a significant increase in NETs production by neutrophils compared to normal human plasma (p<0.05). C: Separated plasma from septic patients stimulated neutrophils from septic patients and normal human neutrophils separately and there was no significant difference in the amount of NETs produced between the two (p >0.05). D: TUNEL to label break DNA (green) and immunofluorescence to label nuclei (blue), with significantly increased levels of break DNA in the nuclei of patients with sepsis compared to controls. E, F: Flow cytometry to detect TUNEL levels in central granulocytes, with significantly increased TUNEL levels in neutrophils of patients compared to controls. I: Immunofluorescence labeling of citH3 (red) with nuclei (blue), increased histone expression in the nuclei of neutrophils in patients compared to controls. H: ELISA assay of histone levels in the plasma of patients with sepsis, significantly increased histone levels in peripheral blood compared to controls.


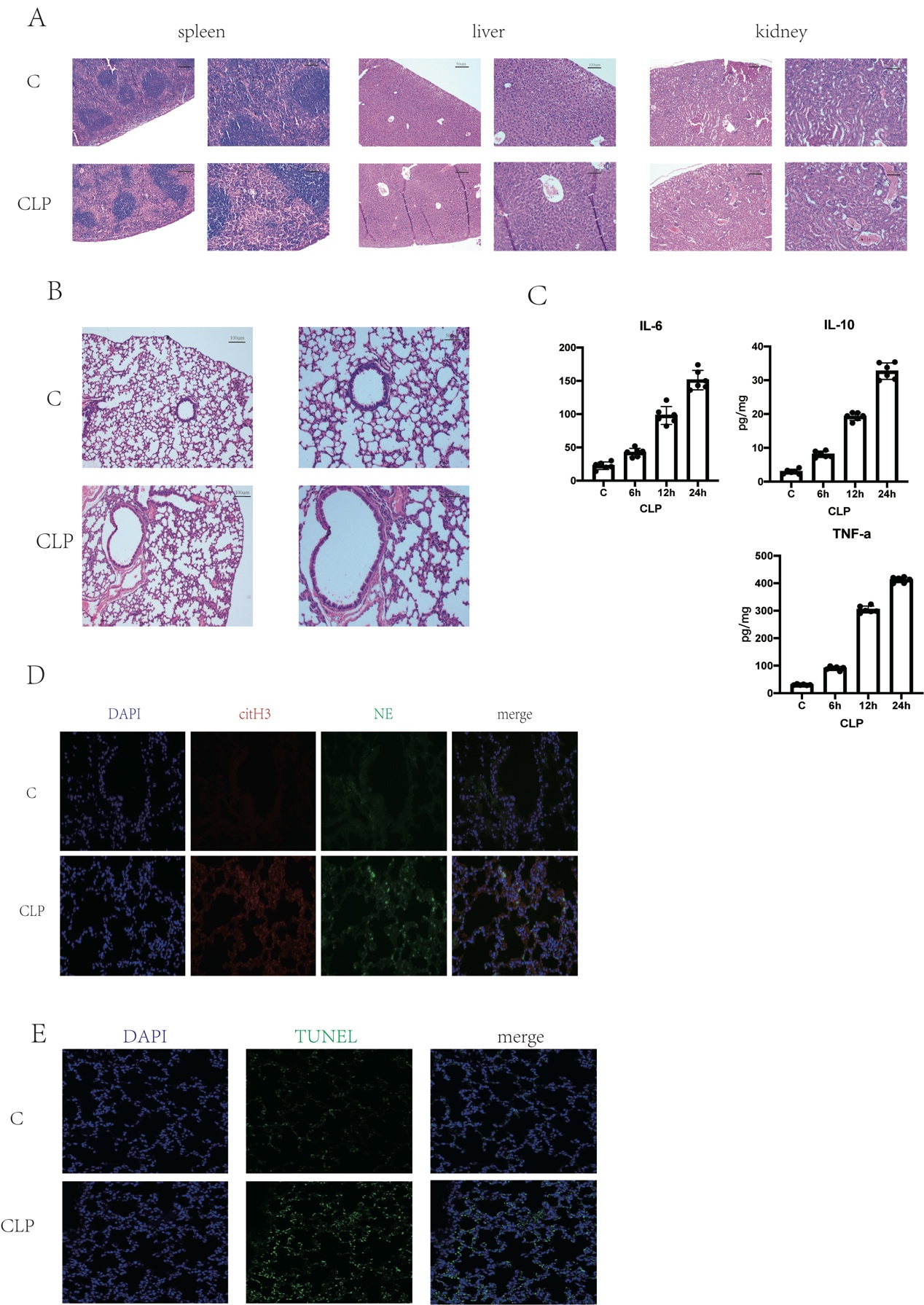


**Figure S2. Massive neutrophils infiltration and NETs formation in the lungs of the CLP model.** A: 24h after modeling of CLP mice, spleen, liver and kidney tissues were significantly edematous compared to the control group. B: 24 hours after the establishment of a mouse model of sepsis, the staining was performed on the lungs, which showed massive destruction of the pulmonary alveoli, and massive cellular infiltration in the lungs of CLP mice compared with normal mice. C: Increased components of inflammatory factors (IL-6, IL-10, TNF-a) in alveolar lavage fluid after modeling of CLP mice. D: Fluorescently labeled lung tissue sections with NE (green), citH3 (red), significantly increased NE and citH3 in lungs of CLP group. E: Immunofluorescence TUNEL was performed on lung tissue, and apoptotic cells were increased in the lungs of septic mice (scale bar: 50 μm).


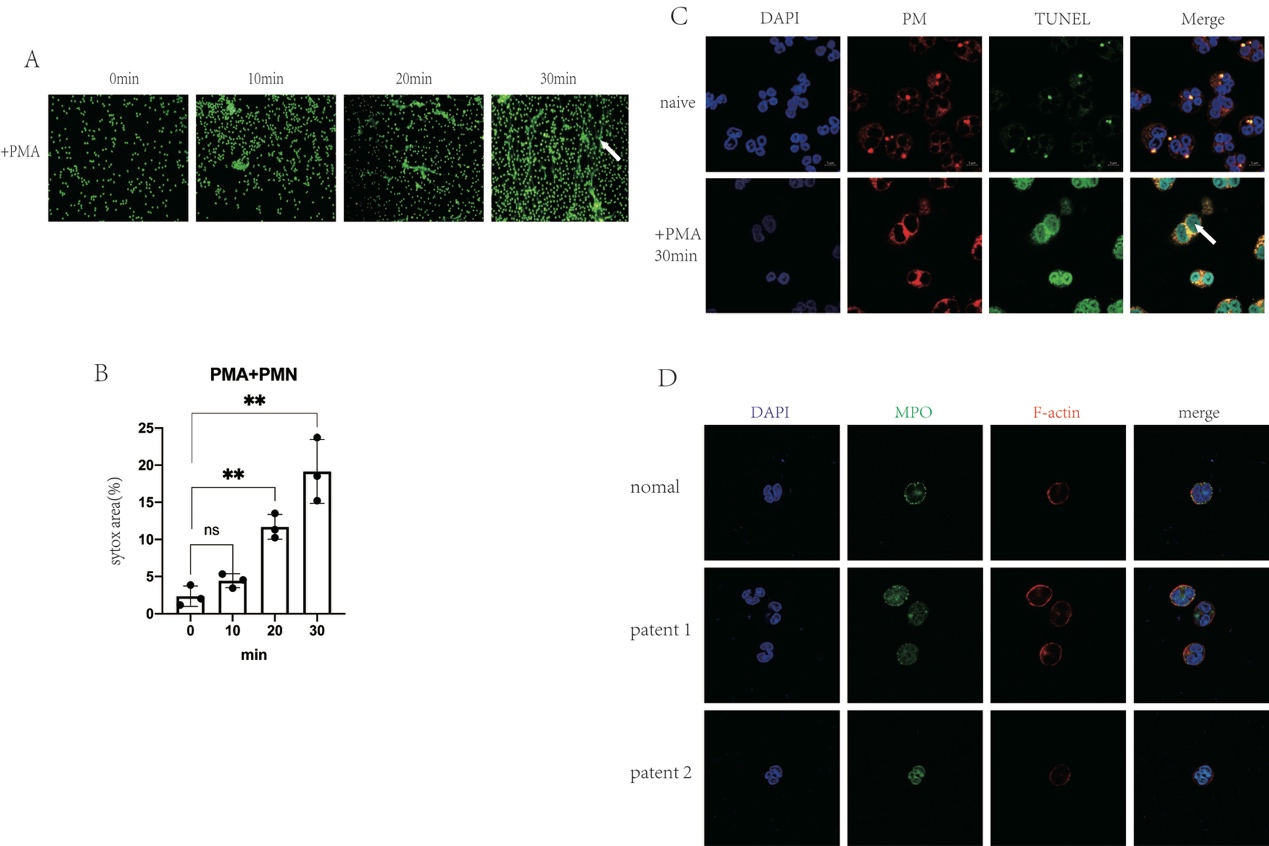


**Figure S3. The nucleus is intrinsically changed early in PMA-stimulated neutrophils.** A: DNA staining using sytox (scale bar: 100 μm) (↑: Increase in NETs generation). B: Analysis of the area of the extracellular DNA (NETs) region using image J. NETs production gradually increased with time but did not exceed 20%. C: TUNEL (green) staining of normal neutrophils and neutrophils activated by PMA for 30 min. large amounts of broken DNA are present in the nucleus of PMA-activated neutrophils, while the cell membrane (red) remains intact (scale bar: 5 μm) (↑: TUNEL staining increased in the nucleus). D: Immunofluorescent labeling of neutrophil MPO (green), nucleus (blue). In healthy neutrophils, MPO is distributed around the nucleus. In contrast, in patients, there are many cells with MPO co-localized with the nucleus (1, 2).


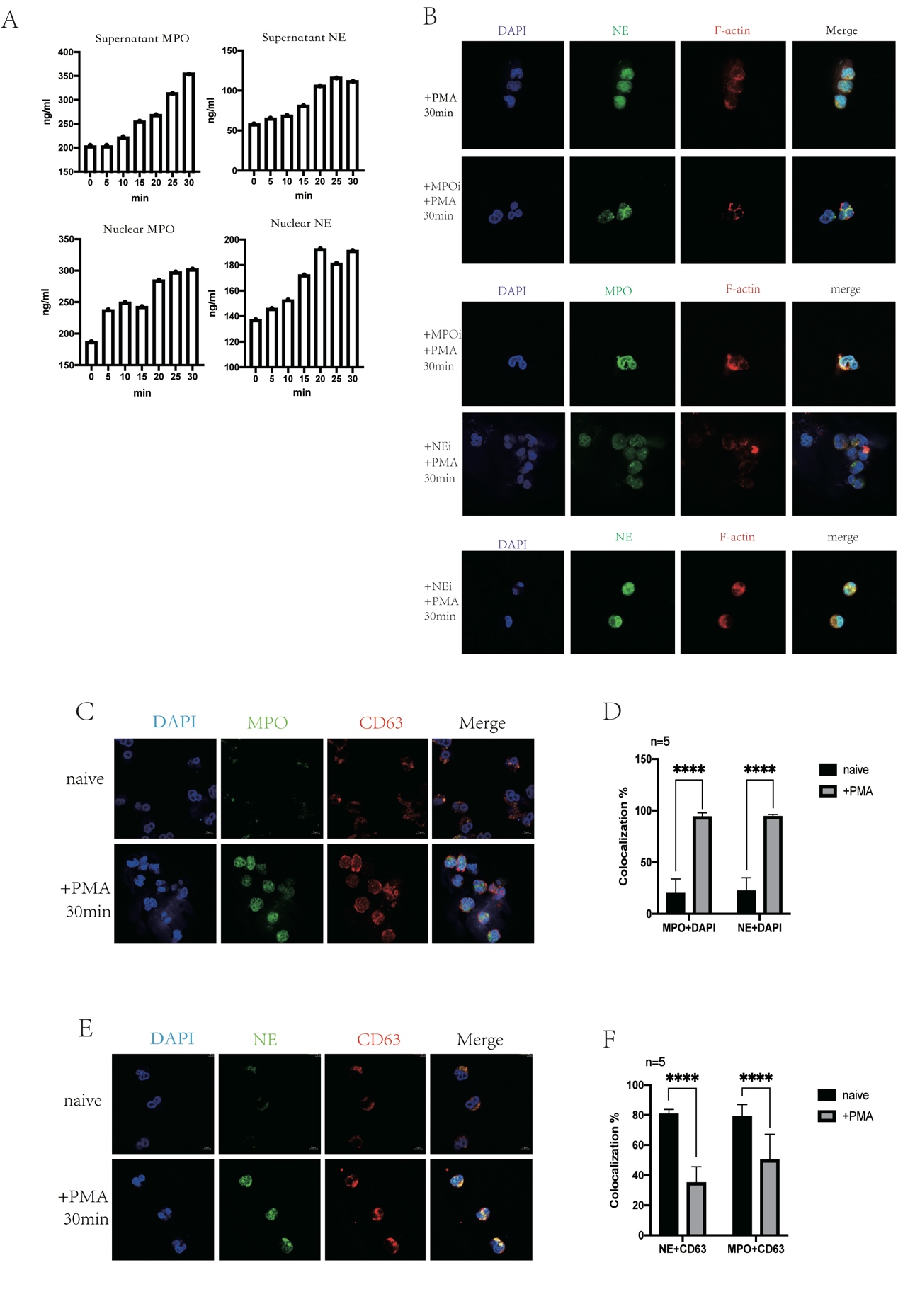


**Figure S4.** **MPO and NE enter the nucleus differently.** A: Intranuclear and extracellular MPO and NE concentrations were measured by ELISA within 30min of PMA stimulation. B: Neutrophils incubated with or without MPOi activated by PMA were fluorescently stained for NE, F-actin, and nuclei, respectively, and MPOi can inhibit NE (green) entry into the nucleus (blue). Pre-incubation of neutrophils with MPOi and NEi, respectively. MPO (green) co-localization with nuclei (blue) increased after PMA stimulation of neutrophils for 30 min. Pre-incubation of neutrophils with NEi and PMA stimulation of neutrophils for 30 min. NE (green) co-localization with the nucleus (blue) increased. (scale bar: 5 μm). C, E: Fluorescent staining of neutrophil nuclei, MPO, NE, CD63, respectively. MPO (C. green), NE (E. green) co-localization with nucleus increased and co-localization with CD63 decreased after PMA stimulation of neutrophils for 30 min (scale bar: 5 μm). D, F: Using image J analysis, MPO(NE) co-localization with the granule was reduced, while increased co-localization with the nucleus


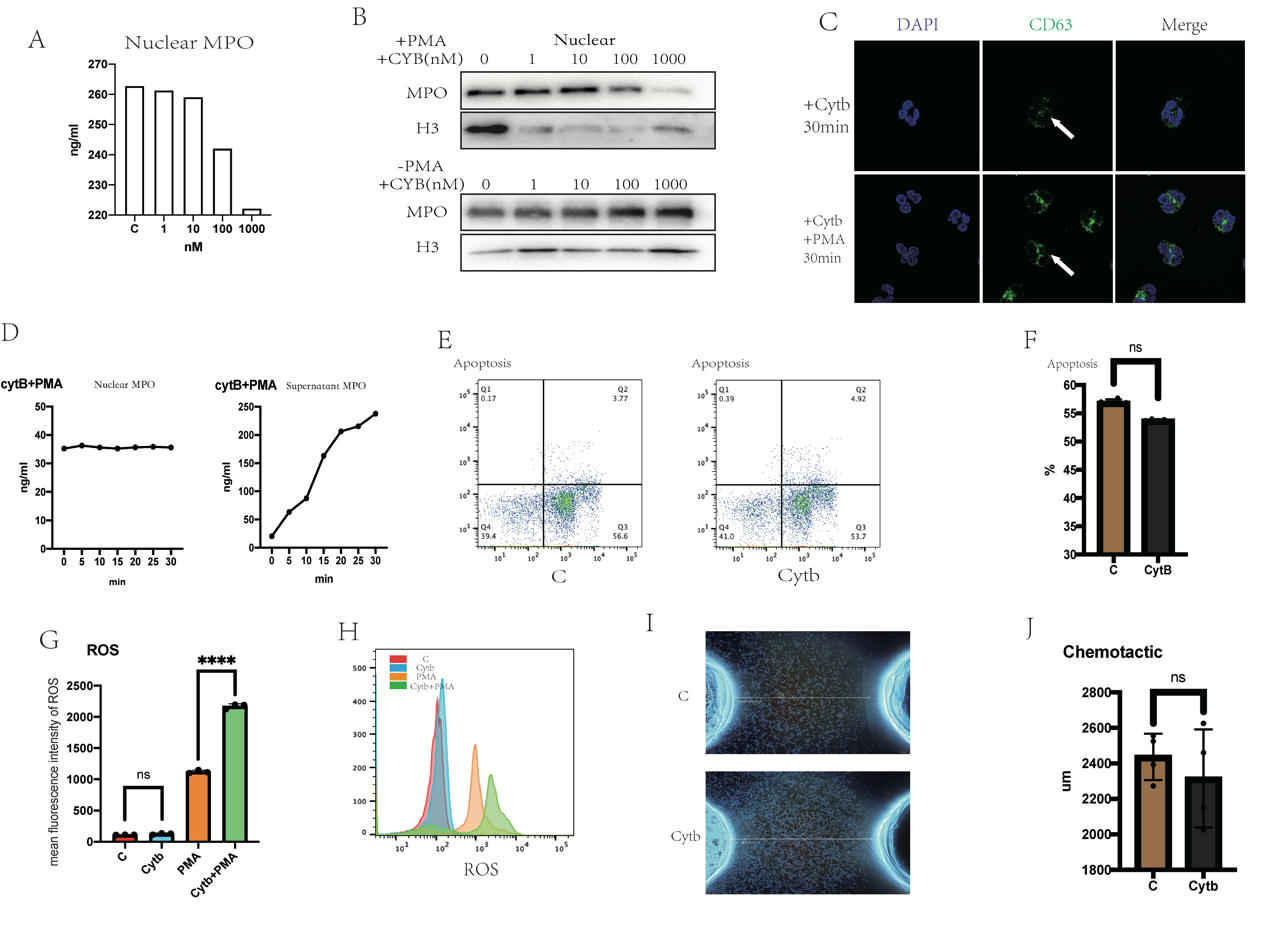


**Figure S5.** **Effect of cytochalasin B on neutrophil function.** Neutrophils were preincubated with different concentrations of CytB for 5 min followed by PMA stimulation of neutrophils for 30 min. Nuclear and cytoplasmic proteins were extracted separately. A: Elisa assay for cytoplasmic and nuclear MPO CytB. B: WB assay for nuclear MPO, histone content and cytoplasmic histone content. C, D: CytB pre-incubation of neutrophils, C: Fluorescent staining of neutrophils for CD63, MPO, F-actin, cell membrane (PM), nuclei (scale bar: 5 μm). PMA stimulation did not result in significant changes in CD63 distribution (↑:CD63 distribution). D: ELISA showed that the nuclear MPO content was low and did not increase over time, and extracellular MPO still increased gradually over time. E: 100nM CytB incubation of neutrophils with flow assay of neutrophil apoptosis rate. F: No significant difference in apoptosis rate between the two groups compared to control (p>0.05). G, H: 100nM CytB pre-incubation of neutrophils with PMA stimulation for 30min, CytB can increase PMA stimulated generation of ROS compared to control. I, J: 100nM CytB pre-incubated neutrophils, no significant difference in chemotactic distance compared to control (p>0.05).

**
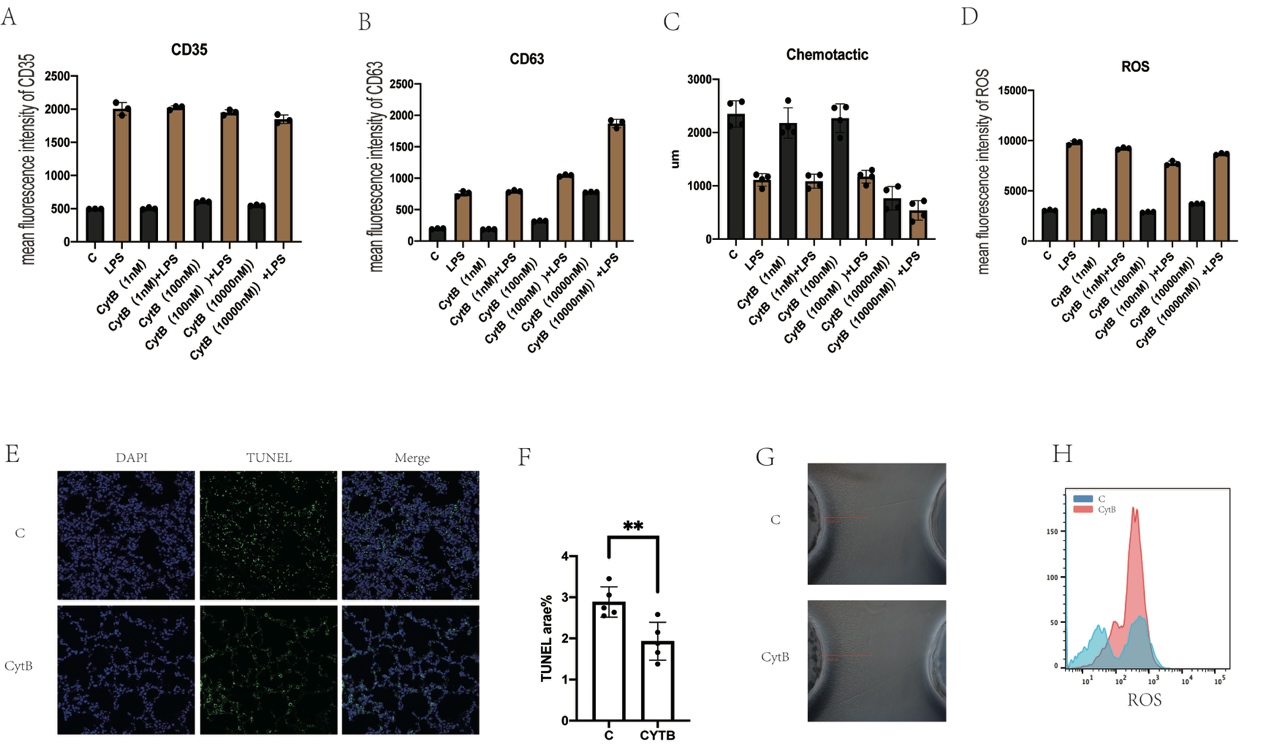
**

**Figure S6. Adequate concentrations do not affect peripheral blood neutrophil function in CLP mice.** Pre-incubation of neutrophils using different concentrations of CytB. A: CytB did not affect secretory vesicle (CD35) degranulation compared to control, and continued stimulation of neutrophils with LPS, CytB also did not affect secretory vesicle (CD35) degranulation. b: 10,000nM CytB increased aspergillus granule (CD63) degranulation compared to control, and continued stimulation of neutrophils with LPS, 10,000nM CytB also increased aspergillus granule (CD63) degranulation. C: 10,000nM CytB significantly decreased neutrophil chemotaxis compared to control, and 10,000nM CytB similarly decreased neutrophil chemotaxis when neutrophils were continued to be stimulated with LPS.D: 10,000nM CytB significantly decreased neutrophil chemotaxis compared to control, and 10,000nM CytB similarly decreased neutrophil chemotaxis when neutrophils were continued to be stimulated with LPS.D: 10,000nM CytB significantly decreased neutrophil chemotaxis compared to control group, CytB did not affect ROS production. E: CLP mice lungs were stained for TUNAL. F: Image j software analysis of TUNAL staining ratios. G: Peripheral blood was taken from CLP mice. Agarose gel model to detect neutrophil chemotaxis. H: Flow cytometry to detect neutrophil ROS production capacity after PMA stimulation of neutrophils.

**Supplemental Movies**


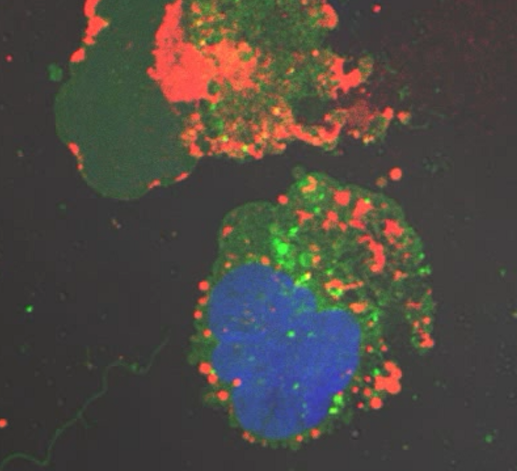


**Movie S1. F-actin undergoes depolymerization/re-polymerization PMA-stimulated neutrophils** Neutrophil nuclei (DAPI, blue), F-actin (red), cell membrane (green) were labeled with fluorescence. Neutrophils were stimulated with PMA and photographed every 20 seconds for 30 minutes. Neutrophil nucleus gradually de-agglutination. F-actin undergoes depolymerization and then re-polymerization. The cell membrane undergoes deformation.


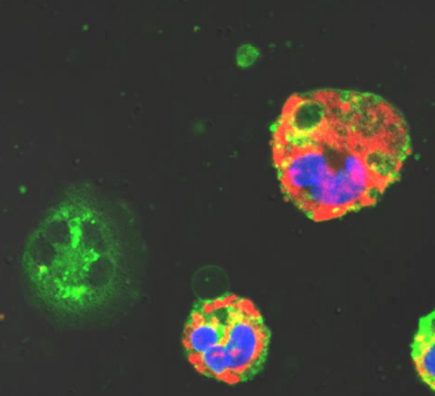

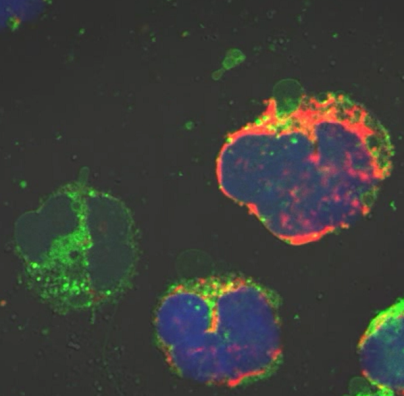

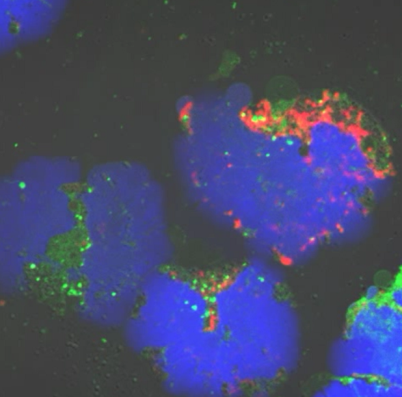


**Movie S2-4.** **NETs formation process in PMA-stimulated neutrophils** Neutrophil nuclei (DAPI, blue), F-actin (red), and cell membrane (green) were labeled with fluorescence. Neutrophil nuclei (DAPI, blue), F-ACTIN (red), cell membrane (green) were labeled with fluorescence. Continuous filming for 90 minutes. Neutrophil nuclei gradually de-agglutination and release to generate NETs. f-actin undergoes depolymerization then re-polymerization. The cell membrane is deformed and ruptured.


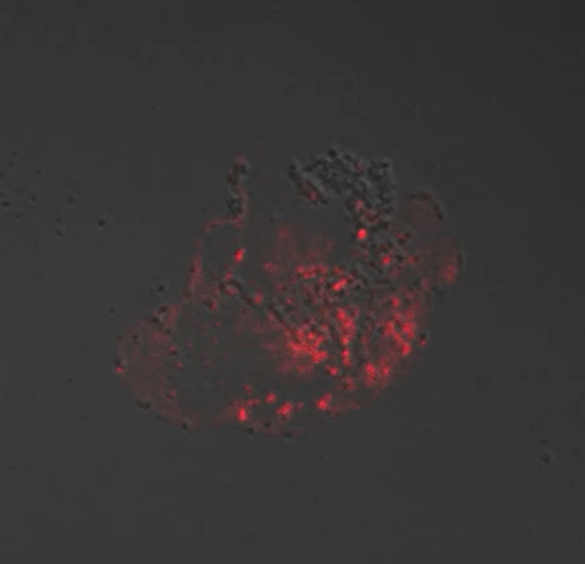


**Movie S5.** **F-actin undergoes re-polymerization at 10-15 min.** Fluorescently labeled neutrophil F-actin, PMA stimulated neutrophils, photographed every 20 s for 30 min. F-actin undergoes re-polymerization at 10-15 min.


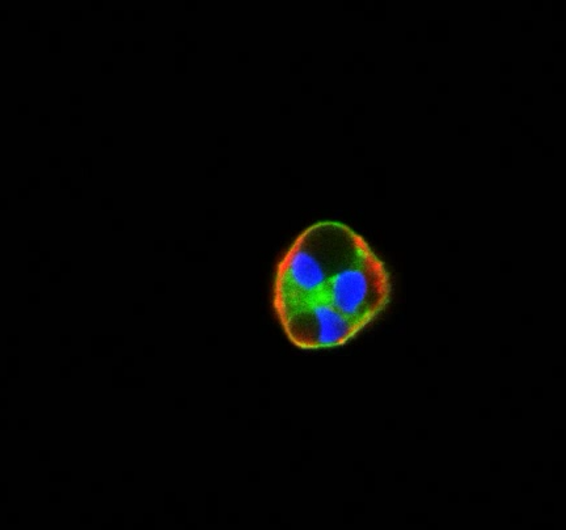


**Movie S6.** **Unstimulated neutrophils.** Neutrophil nuclei (DAPI, blue), F-actin (red), and cell membranes (green) were labeled with fluorescence. F-actin is mainly concentrated under the cell membrane and the cell shape is well maintained.


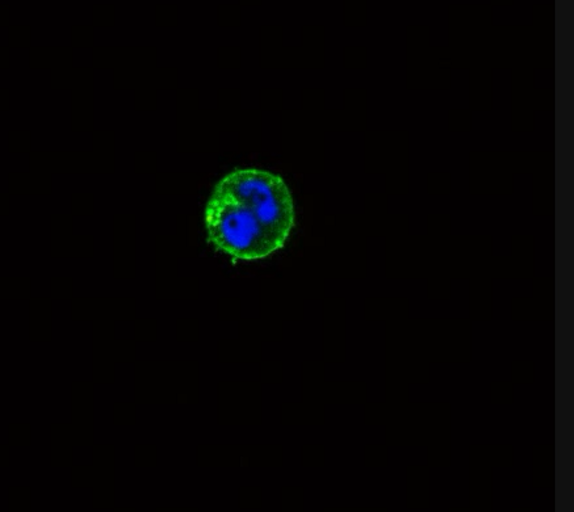


**Movie S7.** **CytB inhibits NETs formation in PMA-stimulated neutrophils** Neutrophil nuclei (DAPI, blue), F-actin (red), and cell membrane (green) were fluorescently labeled. CytB was preincubated for 5 min, and PMA was added to stimulate the neutrophils, and the cells were photographed every 20 s for 30 min. F- actin could not be visualized due to depolymerization, and the nucleus and cell membrane were maintained intact.
